# Association between socio-economic factors and the risk of overweight and obesity among Chinese adults: a retrospective cross-sectional study from the China Health and Nutrition Survey

**DOI:** 10.1186/s41256-022-00274-y

**Published:** 2022-10-31

**Authors:** Kai Wang, Caifeng Wu, Yifan Yao, Shihan Zhang, Yaxuan Xie, Kejian Shi, Zhanpeng Yuan

**Affiliations:** 1 School of Public Health, Wuhan University, Wuchang District, Wuhan, 430071 China; 2 Chengdu Medical College, Xindu District, Chengdu, 610500 China; 3 School of Medicine and Health Management, Tongji Medical College, Huazhong University of Science and Technology, Qiaokou District, Wuhan, 430030 China

**Keywords:** Socio-economic factors, Overweight, Obesity, CHINESE adults, Gender differences

## Abstract

**Background:**

With the rising prevalence of obesity and overweight, increasing number of scholars paid attention to the negative effects on human health and life. Recent years, many studies have focused on the relation of socio-economic factors with the risk of overweight or obesity, but findings have been inconsistent. This study investigated the relationship between socio-economic factors and the risk of overweight and obesity among Chinese adults.

**Methods:**

This study was based on the survey of the China Health and Nutrition Survey in 2015, with 9245 Chinese adults aged 18–65 years old. Overweight and obesity were assessed by physical measurements of weight, height, and waist circumference. Multiple logistic models were used to estimate the odds ratios (ORs) and 95% confidence intervals (CIs) of the association.

**Results:**

Overall, the prevalence rates of general obesity and abdominal obesity were 15.5% and 22.6%, respectively. We found that education and per capita household income were positively associated with overweight and obesity risk in men. However, the association between education and obesity status was negative in women [general obesity: OR = 0.64, 95% CI (0.50–0.81); abdominal obesity: OR = 0.62, 95% CI (0.51–0.76)]. Occupational status was only associated with general overweight in men.

**Conclusions:**

Results suggested that higher education and per capita household income were associated with an increased risk of overweight and obesity among Chinese men, whereas the associations were negative for women. We recommended that men with high levels of education and income, women with low levels of education, can engage in some physical activity, modify dietary, and adopt a new way of life to maintain their weight and general health.

## Background

Overweight and obesity are defined as abnormal or excessive fat accumulation, which can damage health, and has become a major health burden worldwide [1]. Body mass index (BMI) and waist circumference (WC) can be used to assess the overweight and obesity. A report of the WHO indicated that overweight is one of the top 10 global health risk factors around the world, even among the top 5 global health risk factors in developed countries [2]. Since 1980, mean BMI and the prevalence of overweight and obesity have rapidly increased [3, 4]. One in three people is classified as overweight or obesity among the world population [4]. The number of adults suffering obesity has reached 603.7 million by 2015 worldwide [5]. In China, the prevalence of overweight and obesity showed a trend of increase [6]. According to the prediction of the National Health Commission of China (NHCC), from 2002 to 2015, the prevalence of overweight and obesity in China rose from 7.1% and 22.85% to 11.9% and 30.15%, respectively. Not only is obesity linked to mental health disorder such as attention deficit hyperactivity disorder, but also associated with the risk of chronic diseases such as cardiovascular disease, cancer, and type 2 diabetes [7–10]. Furthermore, excess weight can lead to musculoskeletal disorders, especially osteoarthritis, a highly disabling degenerative disease of the joints. It leads to a lower quality of life and general health [11]. Concurrently, overweight and obesity may have a considerable impact on healthcare expenditures. A European study found that obesity appeared to account for a significant economic burden in many European countries. Obesity-related healthcare burdens of up to 10.4 billion euros and the economic burdens ranged from 0.09 to 0.61% of each country's gross domestic product (GDP) [12]. By 2030, it is predicted that obesity-related medical expenses in the United States would reach $48 billion to $66 billion annually [13].

Many studies have focused on the relation of socio-economic factors with the risk of overweight or obesity, but findings have been inconsistent [14–18]. Socio-economic factors mainly include income, education, and occupational status, and such factors, which play an important role in the development of overweight and obesity. Fred C and his colleagues concluded that while economic and social development can improve health, it can also increase obesity and expand socioeconomic status disparities in obesity [19]. A large population-based cross-sectional study involving 991,327 women from lower- and middle-income countries found that socio-economic status was positively associated with the risk of overweight and obesity [17]. A cross-sectional multicenter population-based investigation of Russians reported that education was positively associated with the risk of obesity [14]. On the contrary, a German study showed higher education level was associated with a reduced risk of overweight or obesity [16]. Although several previous studies investigated the association of socio-economic factors with overweight and obesity, the inconsistent results still make these associations unclear.

Therefore, in this study, we aimed to evaluate the association between socio-economic factors with the risk of overweight and obesity among 9245 Chinese adults from a large population-based survey. Furthermore, we conducted analyses stratified by gender to explore whether this association could be affected by gender differences. Our study contributed to the development of healthy living guidelines for the Chinese population, as well as providing some theoretical support for the development of relevant public health prevention policies.

## Methods

### Data source and study population

In this study, we used the data from the China Health and Nutrition Survey (CHNS) in 2015. The CHNS is a population-based cohort study that used a multistage random-cluster sampling process to survey Chinese residents from 15 provinces in China. The selected provinces (autonomous regions) were representative based on the measurement of various factors and the level of social development in China. This survey was designed to evaluate the health and nutritional status of the Chinese population resulting from the social and economic transformation. Thus far, the CHNS has finished 10 rounds of surveys from 1989 to 2015. Details of the investigation have been described elsewhere [20]. All participants signed informed consent forms. The survey was approved by the institutional review committees of the University of North Carolina at Chapel Hill, the National Institute of Nutrition and Food Safety, and the Chinese Center for Disease Control and Prevention.

This study included 10,640 participants aged from 18 to 65 years old. We excluded participants without height and weight measurement, complete information of education level, household income, or occupation (*n* = 1322). Because measurements of WC in pregnant or lactating women have no objective significance, we also excluded those who were pregnant or lactating (n = 73). Multiple imputation or both imputation were used for missing covariates. Finally, 9245 participants (4375 men and 4870 women) were included in this cross-sectional study.

### Assessment of overweight and obesity

In our study, BMI and WC are measured by CHNS, they can be used to assess the overweight and obesity. Physical measurements are made by professionals using uniform equipment. In all surveys, people's height and weight were assessed while wearing loose clothes, and no shoes and hat. Before the measurement, the reel height measurement instrument was adjusted to zero and participants stood with their back against a wall, looking straight ahead and parallel to the floor. The weight was measured by electronic weight scale, which also needed to be calibrated before measurement [21]. BMI is defined as weight (kg) divided by the square of height (m) (kg/m^2^). WC (in cm) was measured with a tape measure at the mid-point between the lower edge of the rib cage and the iliac crest [22]. Body weight was measured to the nearest 0.1 kg, height to the nearest 0.1 cm, and WC to the nearest 0.1 cm [23]. According to the guidelines of the Working Groups on Obesity in China (WGOC) [24], BMI was divided into four groups: underweight (BMI < 18.5 kg/m^2^); normal (BMI ≥ 18.5 kg/m^2^ but < 24 kg/ m^2^); overweight (BMI ≥ 24.0 kg/ m^2^ but < 28 kg/ m^2^); and obesity (BMI ≥ 28 kg/ m^2^). WC was categorized into three groups: normal (WC < 80 cm for women and WC < 85 cm for men); overweight (WC ≥ 80 cm but < 90 cm for women, WC ≥ 85 cm but < 95 cm for men); obesity (WC ≥ 90 cm for women and WC ≥ 95 cm for men).

### Assessment of socio-economic factors

In this study, we collected three socio-economic factors through the questionnaires, namely, education levels, per capita household income, and occupational status. China implemented 9-year compulsory education in 1986, and the age range of the survey population was between 18 and 65 years old. In order to conform to China's national conditions and actual situation, combined with the original questionnaire design, this study divided the education level into the three groups: less than primary school; less than high school; and higher than high school. Per capita household income was calculated by dividing total household income by the number of people in the household [25]. Participants were ranked by their per capita household income from the smallest to the largest, and divided into four quartile spacing groups: < 8000 CNY; ≥ 8000 and < 17,000 CNY; ≥ 17,000 and < 30,800 CNY; ≥ 30,800 CNY. Occupational status was divided into current working or not working.

### Assessment of covariates

Covariates included age, gender (female and male), residence (urban, rural), marital status (never married, married, divorced, or widowed), leisure physical activity (LPA), alcohol intake (never, no more than once a month, once or twice a month, once or twice a week, three or four times a week, almost every day), smoking status (never; former; current), hypertension and type 2 diabetes (T2D). LPA was assessed by multiplying the time an individual spent in each activity (6 items of active activities, 7 items of sedentary activities) by metabolic equivalent (MET) score, which is an indicator of the average intensity of each LPA [26]. Hypertension was ascertained if subjects met at least one of the present criteria: (1) a physician diagnosis of hypertension; (2) antihypertensive treatment; (3) systolic BP ≥ 140 mmHg; (4) diastolic BP ≥ 90 mmHg. T2D was defined as meeting at least one of the following criteria [27, 28] (1) FPG ≥ 7.0 mmol/L; (2) HbA1c ≥ 6.5%; (3) a history of T2D diagnosis; (4) taking any diabetic medication or anti-hyperglycaemic treatment.

### Statistical analysis

Continuous variables were described as means and standard deviations (SDs), and categorical variables were presented as frequency with percentage. We used chi-square test for categorical variables and ANOVA for continuous variables to test linear trends across BMI groups and WC groups, separately.

Multiple logistic regression models were used to estimate the associations with the risk of overweight and obesity of socio-economic factors. The magnitude of the associations was assessed by odds ratios (ORs) and 95% confidence intervals (CIs). We performed two models to examine the association of the socio-economic factors with the overweight and obesity. Model 1 was adjusted for age and residence (urban, rural). Model 2 was adjusted as for basic model and further adjusted for marital status (never married, married, divorced, or widowed), smoking status (never, former, current), alcohol intake (never, no more than once a month, once or twice a week, three or four times a week, almost every day), LPA (categorical variable), history of hypertension type 2 diabetes. All statistical analyses were conducted using SAS version 9.4 (SAS Institute Inc., Cary, NC, USA). Statistical significance was defined by two-sided *p* < 0.05 for all tests.

## Results

### Characteristics of the participants

Our study included 9245 participants, of which 47.3% (N = 4375) were men, and 52.7% (N = 4870) were women. The basic characteristics of the participants is shown in Table 1. Overall, overweight and obesity defined by WC (35.5% and 22.6%) were more prevalent than those defined by BMI (34.3% and 15.5%). Participants with higher BMI or larger WC were more likely to be older, got lower education and higher per capita household income, did less LPA and had higher blood pressure, and had a history of hypertension and type 2 diabetes. Furthermore, participants with higher BMI were more likely to be male and consume more alcohol. Subjects with larger WC were more likely to live in urban areas and be currently working.

**Table 1:** Characteristics for 9245 participants according to body mass index (BMI) and waist circumference (WC)

Characteristics	BMI				*p* trend*	WC			*p* trend*
	Underweight	Normal	Overweight	Obesity		Normal	Overweight	Obesity	
Participants (%)	401 (4.34%)	4283 (46.33%)	3171 (34.30%)	1390 (15.04%)		3880 (41.97%)	3279 (35.47%)	2086 (22.56%)	
Age (year)	38.79 ± 14.40	45.29 ± 12.66	48.52 ± 10.80	47.22 ± 11.94	< 0.001	43.36 ± 13.04	48.05 ± 11.13	49.49 ± 10.94	< 0.001
Male (%)	149 (37.16%)	1939 (45.27%)	1600 (50.46%)	687 (49.42%)	< 0.001	1821 (46.93%)	1568 (47.82%)	986 (47.27%)	0.7545
Urban residence (%)	135 (33.67%)	1584 (36.98%)	1264 (39.86%)	546 (39.28%)	0.0143	1391 (35.85%)	1286 (39.22%)	852 (40.84%)	< 0.001
Marital status (%)					< 0.001				< 0.001
Never married	100 (24.94%)	466 (10.88%)	142 (4.48%)	121 (8.71%)		539 (13.89%)	189 (5.76%)	101 (4.84%)	
Married	292 (72.82%)	3638 (84.94%)	2901 (91.49%)	1205 (86.69%)		3210 (82.73%)	2947 (89.87%)	1879 (90.08%)	
Divorced or widowed	9 (2.24%)	179 (4.18%)	128 (4.04%)	64 (4.60%)		131 (3.38%)	143 (4.36%)	106 (5.08%)	
BMI (kg/m^2^)	17.37 ± 1.28	21.75 ± 1.46	25.79 ± 1.11	31.35 ± 5.42		22.05 ± 3.77	24.90 ± 3.84	27.92 ± 3.55	< 0.001
WC (CM)	68.37 ± 10.59	78.00 ± 11.15	87.23 ± 11.28	94.34 ± 15.96	< 0.001	72.11 ± 12.67	86.62 ± 3.85	98.48 ± 6.49	
Education (%)					< 0.001				< 0.001
≤ Primary school	71 (17.71%)	901 (21.04%)	748 (23.59%)	333 (23.96%)		764 (19.69%)	749 (22.84%)	540 (25.89%)	
≤ High school	134 (33.42%)	1605 (37.47%)	1202 (37.91%)	544 (39.14%)		1461 (37.65%)	1246 (38.00%)	778 (37.30%)	
> High school	196 (48.88%)	1777 (41.49%)	1221 (38.51%)	513 (36.91%)		1655 (42.65%)	1284 (39.16%)	768 (36.82%)	
Per capita household income (CNY) (%)					0.012				< 0.001
< 8000	120 (29.93%)	1097 (25.61%)	748 (23.59%)	342 (24.60%)		1047 (26.98%)	762 (23.24%)	498 (23.87%)	
≥ 8000 to < 17,000	120 (29.93%)	1071 (25.01%)	770 (24.28%)	347 (24.96%)		980 (25.26%)	834 (25.43%)	494 (23.68%)	
≥ 17,000 to < 30,800	81 (20.20%)	1041 (24.31%)	830 (26.17%)	365 (26.26%)		926 (23.87%)	834 (25.43%)	557 (26.70%)	
≥ 30,800	80 (19.95%)	1074 (25.08%)	823 (25.95%)	336 (24.17%)		927 (23.89%)	849 (25.89%)	537 (25.74%)	
Employed people (%)	225 (56.11%)	2504 (58.46%)	1816 (57.27%)	776 (55.83%)	0.3087	2361 (60.85%)	1856 (56.60%)	1104 (52.92%)	< 0.001
Never smoke (%)	306 (76.31%)	3124 (72.94%)	2301 (72.56%)	1033 (74.32%)	0.3016	2816 (72.58%)	2402 (73.25%)	1546 (74.11%)	0.4381
No alcohol intake (%)	295 (73.57%)	2964 (69.20%)	2166 (68.31%)	923 (66.40%)	< 0.001	2664 (68.66%)	2239 (68.28%)	1445 (69.27%)	0.0926
Leisure physical activity (MET-h/week)	32.55	27.30	24.85	26.18	< 0.001	27.30	26.53	24.59	< 0.001
Hypertension (%)	41 (10.22%)	824 (19.24%)	1135 (35.79%)	668 (48.06%)	< 0.001	661 (17.04%)	1035 (31.56%)	972 (46.60%)	< 0.001
Type 2 diabetes (%)	3 (0.75%)	84 (1.96%)	131 (4.13%)	81 (5.83%)	< 0.001	60 (1.55%)	109 (3.32%)	130 (6.23%)	< 0.001
Systolic blood pressure (mm Hg)	115.22 ± 16.05	122.21 ± 16.52	129.20 ± 16.90	134.39 ± 18.03	< 0.001	120.84 ± 16.38	127.48 ± 16.81	133.50 ± 17.83	< 0.001
Diastolic blood pressure (mm Hg)	75.15 ± 10.31	78.57 ± 9.99	83.21 ± 10.55	86.83 ± 11.44	< 0.001	78.07 ± 10.11	82.24 ± 10.52	85.44 ± 11.25	< 0.001

Data are presented as mean value and SD for continuous variables and percentages for categorical variables. Leisure physical activity was presented as median

*Linear trends across BMI and WC quintiles were tested using ANOVA for continuous variables, Chi-square test for categorical variables

### The association between education levels and the risk of overweight and obesity

**Fig. 1:**
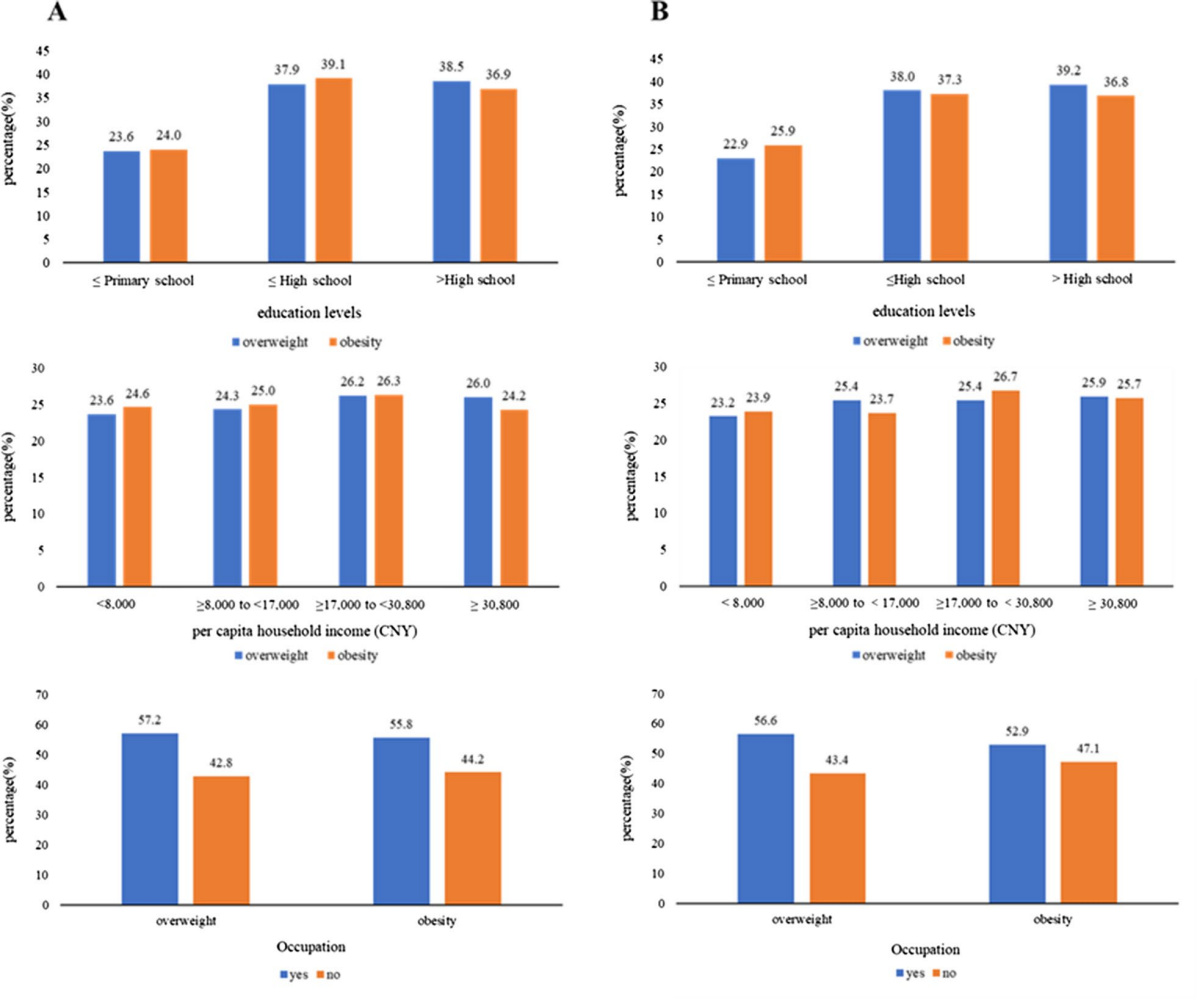
Percentages of overweight and obesity with different education levels, income groups, occupation status (A: BMI, B: WC). *Note*: BMI is defined as weight (kg) divided by the square of height (m) (kg/m^2^), WC (in cm) wasmeasured with a tape measure at the mid-point between the lower edge of the rib cage and the iliac crest

The proportion of overweight and obesity in the different education levels is gradually increasing, and the education level of the largest proportion of people is high school level and above (Fig. 1). The association between the risk of overweight and obesity with education level were significant in men and women. In model 1, we adjust age and residence, and the results showed that higher education level was positively associated with an increased risk of general overweight and obesity in men [overweight: OR (95% CI) 1.33 (1.09–1.64); obesity: OR (95% CI) 1.65 (1.22–2.22)] (Table 2), whereas the associations were negative in women [overweight: OR (95% CI) 0.76 (0.63–0.92); obesity: OR (95% CI) 0.52 (0.40–0.66)] (Table 3). The associations were opposite across genders. In men, the magnitude of the associations weakened after additionally adjusted for health-related factors and history of hypertension and T2D. However, the associations remained stable in women. The relation between education level and abdominal obesity status was also different according to gender. In men, the ORs and 95% CIs of overweight and obesity were respectively 1.20 (0.98–1.47) and 1.42 (1.14–1.76), and 1.36 (1.06–1.74) and 1.77 (1.36–2.29) comparing each group of education level with the lowest level. In women, education level was inversely associated with abdominal obesity [OR (95% CI) 0.62 (0.49–0.79)], whereas no association of education with abdominal overweight risk was observed [OR (95% CI) 0.88 (0.72–1.07].

**Table 2:** Odds ratios (ORs) with 95% confidence intervals (CIs) of socio-economic factors related to overweight and obesity in men (*n* = 4375)

	General obesity status				Abdominal obesity status			
	Overweight		Obesity		Overweight		Obesity	
	Model 1^a^	Model 2^b^	Model 1^a^	Model 2^b^	Model 1^a^	Model 2^b^	Model 1^a^	Model 2^b^
Education level								
≤ Primary school	1.00	1.00	1.00	1.00	1.00	1.00	1.00	1.00
≤ High school	1.24 (1.02–1.50)*	1.20 (0.98–1.46)	1.66 (1.25–2.19)**	1.60 (1.20–2.13)**	1.25 (1.03–1.53)*	1.20 (0.98–1.47)	1.45 (1.14–1.85)**	1.36 (1.06–1.74)*
> High school	1.33 (1.09–1.64)**	1.28 (1.04–1.58)*	1.65 (1.23–2.22)**	1.58 (1.16–2.13)**	1.46 (1.18–1.80)**	1.42 (1.14–1.76)**	1.82 (1.41–2.34)**	1.77 (1.36–2.29)**
*p* trend	0.008	0.023	0.006	0.016	0.001	0.001	< 0.001	< 0.001
Per capita household income (CNY) (%)								
< 8000	1.00	1.00	1.00	1.00	1.00	1.00	1.00	1.00
≥ 8000 to < 17,000	1.17 (0.97–1.41)	1.18 (0.97–1.42)	1.08 (0.84–1.39)	1.09 (0.84–1.41)	1.26 (1.04–1.53)*	1.27 (1.05–1.55)*	1.29 (1.02–1.62)*	1.30 (1.03–1.65)*
≥ 17,000 to < 30,800	1.44 (1.19–1.74)**	1.49 (1.23–1.81)**	1.31 (1.02–1.68) *	1.40 (1.08–2.13)*	1.37 (1.12–1.66)**	1.41 (1.16–1.72)**	1.48 (1.18–1.86)**	1.58 (1.25–2.00)**
≥ 30,800	1.47 (1.21–1.78)**	1.49 (1.22–1.82)**	1.43 (1.10–1.84)**	1.47 (1.13–1.92)**	1.42 (1.16–1.74)**	1.46 (1.18–1.79)**	1.67 (1.32–2.11)**	1.74 (1.37–2.22)**
*p* trend	< 0.001	< 0.001	0.002	0.001	0.001	0.000	< 0.001	< 0.001
Occupation (%)								
No	1.00	1.00	1.00	1.00	1.00	1.00	1.00	1.00
Yes	1.21 (1.05–1.40)**	1.23 (1.06–1.42) **	1.35 (1.11–1.64)**	1.45 (1.18–1.78)* *	1.18 (1.02–1.37)*	1.16 (0.99–1.35)	1.22 (1.03–1.45)*	1.23 (1.02–1.47)*
*p* trend	0.008	0.007	0.002	< 0.001	0.028	0.062	0.022	0.028

*p < 0.05, **p < 0.01

^a^With adjustments for age and residence

^b^With adjustments for age, residence, marital status, smoking status, alcohol intake, LPA, history of hypertension, and history of type 2 diabetes

**Table 3:** Odds ratios (ORs) with 95% confidence intervals (CIs) of socio-economic factors related to overweight and obesity in women (*n* = 4870)

	General obesity status				Abdominal obesity status			
	Overweight		Obesity		Overweight		Obesity	
	Model 1^a^	Model 2^b^	Model 1^a^	Model 2^b^	Model 1^a^	Model 2^b^	Model 1^a^	Model 2^b^
Education level								
≤ Primary school	1.00	1.00	1.00	1.00	1.00	1.00	1.00	1.00
≤ High school	0.96 (0.81–1.13)	0.98 (0.82–1.16)	0.81 (0.65–1.00)*	0.81 (0.65–1.00)	1.03 (0.86–1.23)	1.05 (0.87–1.25)	0.91 (0.75–1.11)	0.94 (0.77–1.15)
> High school	0.76 (0.63–0.92)**	0.78 (0.64–0.95)*	0.52 (0.40–0.66)**	0.54 (0.41–0.70)**	0.86 (0.70–1.06)	0.88 (0.72–1.07)	0.60 (0.48–0.75)**	0.62 (0.49–0.79)**
*p* trend	0.003	0.010	< 0.001	< 0.001	0.090	0.150	< 0.001	< 0.001
Per capita household income (CNY) (%)								
< 8000	1.00	1.00	1.00	1.00	1.00	1.00	1.00	1.00
≥ 8000 to < 17,000	0.98 (0.82–1.18)	0.99 (0.83–1.19)	1.01 (0.80–1.28)	1.01 (0.80–1.29)	1.14 (0.95–1.37)	1.15 (0.95–1.39)	0.96 (0.77–1.19)	0.96 (0.77–1.20)
≥ 17,000 to < 30,800	1.02 (0.85–1.23)	1.03 (0.85–1.24)	1.01 (0.80–1.28)	1.01 (0.79–1.29)	1.16 (0.96–1.41)	1.17 (0.97–1.42)	1.14 (0.92–1.42)	1.14 (0.92–1.43)
≥ 30,800	0.88 (0.73–1.06)	0.91 (0.75–1.10)	0.69 (0.54–0.90)**	0.72 (0.55–0.93)*	1.08 (0.89–1.31)	1.11 (0.91–1.35)	0.84 (0.67–1.05)	0.87 (0.69–1.10)
*p* trend	0.257	0.411	0.012	0.025	0.421	0.290	0.365	0.552
Occupation (%)								
No	1.00	1.00	1.00	1.00	1.00	1.00	1.00	1.00
Yes	1.05 (0.91–1.20)	1.04 (0.91–1.20)	0.79 (0.66–0.95)*	0.80 (0.67–0.97)*	0.92 (0.80–1.06)	0.92 (0.80–1.06)	0.81 (0.69–0.96)*	0.82 (0.69–0.97)*
*p* trend	0.518	0.567	0.011	0.021	0.248	0.230	0.012	0.018

*p < 0.05, **p < 0.01

^a^With adjustments for age and residence

^b^With adjustments for age, residence, marital status, smoking status, alcohol intake, LPA, history of hypertension, and history of type 2 diabetes

### The association between per capita household income and the risk of overweight and obesity

In all samples, those in the ≥ 17,000 and < 30,800 CNY income group had the largest percentage of overweight and obesity (general overweight: 26.2%; general obesity: 26.3%; abdominal overweight: 25.4%; abdominal obesity: 26.7%) (Fig. 1). The association between per capita household income with the risk of overweight and obesity was different for men and women. In men, per capita household income was significantly associated with an increased risk of general overweight and obesity. With additional adjustments in the multivariable model, the association remained significant [overweight: OR (95% CI) 1.49 (1.22–1.82); obesity: OR (95% CI) 1.47 (1.13–1.92)]. In women, no significant associations were found between per capita household income and the risk of general overweight. While the highest income group showed a reduced risk associated with general obesity [OR (95% CI) 0.69 (0.54–0.90)]. In men, a significant linear trend was observed between increased income and abdominal obesity risk (*p* < 0.001). In women, no significant associations were found between per capita household income and the risk of abdominal overweight or obesity. The highest income group showed approximately 10%, albeit not statistically significant, risk reduction associated with abdominal obesity [OR (95% CI) 0.87 (0.69–1.10)].

### The association between occupational status and the risk of overweight and obesity

As to occupational status, people in current working status are more likely to be overweight and obesity (general overweight: 57.2%; general obesity: 55.8%; abdominal overweight: 56.6%; abdominal obesity: 52.9%) (Fig. 1). Results in men showed that there is an association between employed and general/abdominal overweight and obesity. Especially in the basic model, the association between male general overweight and obesity is positive [overweight: OR (95% CI) 1.21 (1.05–1.40); obesity: OR (95% CI) 1.35 (1.11–1.64)]. However, in the multilevel model that adjusts for confounding factors, such association becomes insignificant. Employed men were more likely to be general/abdominal obesity compared to those unemployed [general obesity: OR (95% CI) 1.45 (1.18–1.78); abdominal obesity: OR (95% CI) 1.23 (1.02–1.47)]. In women, occupational status was inversely associated with obesity [general obesity: OR (95% CI) 0.80 (0.67–0.97); abdominal obesity: OR (95% CI) 0.82 (0.69–0.97)], whereas no association of occupational status with the risk of overweight.

## Discussion

In this population-based cross-sectional study of Chinese adults, the association between socio-economic factors and the risk of overweight and obesity differed by gender. Education level was positively associated with the risk of overweight and obesity in men, whereas the results were opposite to women. In men, higher per capita household income was significantly associated with an increased risk of general overweight, abdominal overweight and abdominal obesity. In women, per capita household income was associated with a reduced risk of general obesity and abdominal overweight/obesity, albeit not statistically significant. A positive association between occupational status and general obesity was observed in men, while such association was not found in women.

Results showed that higher education level was positively associated with the risk of overweight and obesity in men, whereas inverse associations were observed in women. Education was a well-known factor of obesity development. Thus far, many studies have evaluated the relationship between education and obesity status. However, the findings have been inconsistent. An Indian cross-sectional study found that higher education level was associated with the risk of overweight and obesity in men and women [29], whereas a Chinese study proposed an inverse association between education and weight [30]. Moreover, a representative population-based study on Burmese population did not find a significant association between education level and the risk of overweight or obesity [31]. This study found that the association between education level and obesity status was different from men and women. Several previous studies showed similar results to our findings. In a review including 333 studies on the relation between socio-economic status and obesity, 63% of the studies found that socio-economic status was negatively associated with obesity risk in women whilst only 37% of these studies found such association in men [32]. A study on the Chinese population reported that higher education level was associated with an increased risk of obesity in men, whereas education was found to be associated with a reduced obesity risk in women [33]. However, findings of some studies were opposite to current study [34, 35]. There were several possible reasons to explain the opposite results for men and women. The sociology of Bourdieu and his theory elaborated on sex differences in body size [36]. For women, those with higher education levels are more likely to get a thinner body, which may be socially valued and materially viable to a greater extent. For men, larger body size is likely to be valued as a sign of physical dominance and prowess. In other words, women pay more attention to physical beauty than men do. Compared with men, women with higher education level are more likely to adhere to a healthier diet, characterized by consuming more of a variety of food and thus have higher quality diets [37].

We found higher per capita household income was associated with an increased risk of overweight and obesity in participants. Two previous studies were in line with our results [30, 38]. A study conducted in rural southwest China reported that household income was positively associated with the prevalence of central obesity [30]. Another study in a rural Han Chinese supported the results of the current study [38]. However, a study involving Tianjin residents found that higher income was associated with a reduced risk of overweight and obesity [33], which is totally opposite to the current finding. A review indicated that the impact of income on weight might follow an inverted U-shape [39]. A possible reason of the current findings was that men with higher income in developing countries were more likely to consume energy dense foods, do a sedentary job, and have few physical activities; all were risk factors related to overweight and obesity. There was a lack of comparability between the results of previous studies and the current study because the study population and regional development level were different in various studies.

Occupational status was associated with the risk of general obesity in men whilst no significant association was noted in women. Thus far, there is no consistent conclusion about the impact of occupation on overweight or obesity. Sedentary works comprise a major part of jobs today [40]. That kind of job would take a long sedentary time and reduce the time of physical activity resulting in weight gain. Physical activity is composed of three main components: occupational activity, household activity such as gardening, cleaning and food preparation; and leisure time activity [41]. However, this study did not include traffic time, or sedentary time, which might result in bias of current finding. Furthermore, the current study categorized occupational status as current working or not working. This classification was different from some previous studies that categorized it into specific types of job. Accurate classification of occupational status was needed in future study to increase comparability between studies.

This study has several strengths, including a representative population-based Chinese sample, and we adjusted for potential confounding factors in models. At the same time, we used the multiple logistic models to analyze the association from a gender discrepancy perspective, to reduce the potential impact of gender differences. Despite the innovations and strengths of this study, the study also has several limitations. First, our study is the cross-sectional design, which is inadequate to confirm the causal association between socio-economic factors and the risk of overweight and obesity. Second, the results may be affected by other factors, such as synergy of genetic inheritance, lifestyle or potential residual confounding factors. Third, our study did not collect dietary data, which is an important factor for obesity development, future research can further incorporate these aspects, and with prudent design is warranted to verify these findings.

## Conclusions

The study revealed that the association between the prevalence of overweight and obesity and socio-economic factors. The results of this study provided important epidemiological evidence for the prevention of overweight and obesity, and can provide a reference for the further research in the future. In view of the serious phenomenon of overweight and obesity and the results of this paper, the following two opinions are put forward to prevent the occurrence of overweight and obesity in the future. First, we should energetically develop health knowledge publicity and sports undertakings. Secondly, we should make progress on social medical and health services. And we also recommended that men with high levels of education and income, women with low levels of education, can do some physical exercises, adjust dietary and change lifestyle to maintain their weight levels and health.

## Data Availability

All data generated or analysed during this study are included in this manuscript.

## Abbreviations

BMI: Body mass index
WC: Waist circumference
NHCC: National Health Commission of China
CVD: Cardiovascular disease
GDP: Gross domestic product
CHNS: China Health and Nutrition Survey
WGOC: Working Groups on Obesity in China
LPA: Leisure physical activity
T2D: Type 2 diabetes
MET: Metabolic equivalent
SD: Standard deviation
OR: Odds ratio
CI: Confidence interval
